# Pickering Emulsions as Vehicles for Bioactive Compounds from Essential Oils

**DOI:** 10.3390/molecules27227872

**Published:** 2022-11-15

**Authors:** Yana Cahyana, Yunita Safriliani Eka Putri, Dian Siti Solihah, Farrah Shabira Lutfi, Randah Miqbil Alqurashi, Herlina Marta

**Affiliations:** 1 Departement of Food Technology, Universitas Padjadjaran, Bandung 45363, Indonesia; 2 Department of Food and Nutrition, College of Agriculture and Food Sciences, King Faisal University, Al-Ahsa 31982, Saudi Arabia

**Keywords:** essential oil, Pickering emulsion, bioactive, antioxidant, antimicrobial, vehicle

## Abstract

Pickering emulsions are emulsion systems stabilized by solid particles at the interface of oil and water. Pickering emulsions are considered to be natural, biodegradable, and safe, so their applications in various fields—such as food, cosmetics, biomedicine, etc.—are very promising, including as a vehicle for essential oils (EOs). These oils contain volatile and aromatic compounds and have excellent properties, such as antifungal, antibacterial, antiviral, and antioxidant activities. Despite their superior properties, EOs are prone to evaporation, decompose when exposed to light and oxygen, and have low solubility, limiting their industrial applications. Several studies have shown that EOs in Pickering emulsions displays less sensitivity to evaporation and oxidation, stronger antibacterial activity, and increased solubility. In brief, the application of Pickering emulsions for EOs is interesting to explore. This review discusses recent progress in the application of Pickering emulsions, particularly as EO carriers, drug carriers, antioxidant and antimicrobial carriers, and in active packaging.

## 1. Introduction

Essential oils (EOs) are categorized as bioactive compounds possessing beneficial properties, such as antibacterial, antifungal, antiviral, antioxidant, anti-inflammatory, and even insecticidal activities. The content and composition of bioactive compounds in essential oils are very diverse and depend on the source of the essential oils [1].

Although EOs possess beneficial properties, in their natural form they are sensitive to environmental influences, which can lead to their degradation and the loss of their beneficial properties. Another drawback of EOs is the hydrophobic nature, which limits their application—especially for food and pharmaceutical purposes [2].

Currently, the trend of using Pickering emulsions is increasing in an effort to develop emulsion systems that are safe for health and the environment. Pickering emulsions, which are particle-stabilized emulsions, are considered to be safer than surfactants—especially when used for medical purposes [3]. Particles commonly used as Pickering emulsion stabilizers are very diverse, including chitosan, starch, cellulose, etc. [4].

Pickering emulsions are known as carriers for bioactive compounds [5]. The role of Pickering emulsions as vehicles for bioactive compounds presents an opportunity to maximize the potential application of essential oils. Edible films, food products, and microcapsules are some examples of products resulting from such emulsion systems. The Pickering emulsion system is expected to be a solution for essential oils that are hydrophobic and prone to oxidation. This method allows EOs to provide a better effect on the resulting product and maintain the benefits of their bioactive components. Given the potential benefits of incorporating EOs into emulsion systems, it is important to provide a review of this application. Therefore, this review aims to explore the potential role of Pickering emulsions as vehicles for bioactive compounds from EOs in various applications.

## 2. Pickering Emulsions

In general, according to their emulsifier forms, emulsion systems can be divided into molecular-emulsifier-based emulsions and particle-emulsifier-based emulsions, also known as Pickering emulsions [6]. Molecular emulsifiers, which are surface-active molecules, are absorbed onto the oil–water interface, leading to the reduction in the interfacial tension and the formation of a protective coating. Strong repulsive forces, such as steric and electrostatic forces generated from the emulsifier molecule layer on the oil–water interface, inhibit droplet aggregation. In this system, absorbed molecular emulsifiers are in equilibrium with non-absorbed ones. On the other hand, in a Pickering emulsion, particles that are partially wetted by both the oil and water phases are irreversibly absorbed onto the interface without significantly decreasing the interfacial tension. These particles form a mechanical barrier against droplet coalescence [6,7].

Particles commonly used in Pickering emulsions include polysaccharide groups, proteins, and other solid particles [8]. Particles used in polysaccharide-based Pickering emulsions include starch, starch nanocrystals, chitosan, cellulose nanocrystals, cellulose nanofibrils, and β-cyclodextrin, while those in protein-based Pickering emulsions include whey, zein, etc. [9]. Other particles commonly used as Pickering emulsion stabilizers include silica nanoparticles, synthetic hydroxyapatite (HAp) nanoparticles, and TiO_2_ nanoparticles.

Pickering emulsions’ stability is influenced by wettability, oil type and volume fraction, particle concentration, pH, and ionic strength. The wettability of the particles determines the type of Pickering emulsion. Particles with a contact angle of 15° < θ < 90° are suitable for *o/w* emulsions, whereas particles with contact angles in the range of 90° < θ < 165° are suitable for *w/o* emulsions.

The type of oil affects the emulsion’s stability because it determines the interaction with the particles as well as the oil–water interface. The volume fraction, which is an oil/water ratio, also affects the emulsion’s stability, where an oil/water ratio above 0.5 leads to a more stable emulsion [10].

The concentration of particles can affect the size of the droplet emulsion. The higher the particle concentration, the smaller the droplet size, resulting in a more stable emulsion [11]. The pH and ionic strength affect the particle charge and the continuous phase conductivity of the emulsion. The charge of the particle can be determined from the zeta potential value. The higher the zeta potential, the smaller the potential for oil droplets to coalesce due to the repulsive force [12]. Therefore, particles with high potency can produce more stable emulsions [13].

## 3. Essential Oils

Essential oils are the liquid extracts produced by plants through the mechanism of secondary metabolite synthesis. Plant parts such as leaves, roots, flowers, stems, and rhizomes are the sources from which EOs can be extracted. EOs contain 20–60 components with different concentrations, including active compounds from the terpene group, alcohols, aldehydes, ketones, esters, ethers, phenols, and many more [14]. EOs, which are regarded as natural compounds, are preferable to synthetic chemical compounds because of their environmentally friendly properties and their safer effects on the body when applied to food and medicine [15]. Some examples of essential oils along with their bioactive contents and functions can be seen in Table 1.

The compounds commonly found in essential oils are characterized as having low molecular weight and can be categorized into two distinct groups: the first is the group of terpenes and terpenoids, while the second constitutes aromatic and aliphatic group [16,17]. Major components (two or three compounds) present at high concentrations in essential oils (20–70%) govern the biological properties of the essential oils [18]. For instance, five major compounds found in essential oil from lemon grass are geranial, neral, myrcene, geraniol, and verbenol [19]. Major compounds found in essential oils from different sources are tabulated in Table 1.

Compounds from the groups of terpenes, as well as ketones (e.g., β-myrcene, α-thujone, geranyl acetate) and phenols (e.g., cinnamaldehyde, carvacrol, eugenol, or thymol) have been identified to possess antibacterial activities [20]. Gram-positive bacteria are more susceptible to the essential oils than Gram-negative bacteria. This may be linked to the different constituents of the cell wall, which hinders the diffusion of essential oil [21]. In general, essential oils show antifungal, antibacterial, and antiviral activity, with the modes of action including membrane integrity breakdown, mitochondrial membrane disintegration, disruption of cellular activity (e.g., energy production and membrane transport), etc. [22]. The hydrophobic nature or essential oils facilitates their diffusion into the lipid layer of the cell membrane.

**Table 1:** Essential oils along with their bioactive contents and functions.

Essential Oil	Scientific Name	Bioactive Compounds	Functions	Ref(s).
Lemon grass	*Cymbopogon citratus,* *Cymbopogon flexuosus*	Geranial, neral, myrcene, geraniol, verbenol	Antibacterial agent, insecticidal agent	[15,19]
Eucalyptus	*Eucalyptus globules*	1,8-cineole, citronellal, citronellol, limonene, α-pinene, α-terpinene	Insecticidal agent	[15]
Rosemary	*Rosmarinus officinalis*	1,8-cineole, camphor, β-caryophyllene, α-terpineol, verbenone	Antibacterial agent, antioxidant, insecticidal agent	[15,19,23]
Clove	*Syzigium aromaticum*	Eugenol, β-caryophyllene, eugenyl acetate, α-humulene	Antibacterial agent, antioxidant	[14,19,24]
Thyme	*Thymus vulgaris*	γ-Terpinene, thymol, p-cymene, carvacrol, linalool	Antibacterial agent, antioxidant, insecticidal agent	[15,19,25]
White peppermint	*Mentha piperita*	Menthol, menthone, menthyl acetate, 1,8-cineole	Antibacterial agent, insecticidal agent	[14,15,19]
Basil	*Ocimum basilicum*	Linalool, 1,8-cineole, geraniol, eugenol	Antibacterial agent, antioxidant, insecticidal agent	[15,19,26]
Oregano	*Origanum vulgar*	Thymol, carvacrol, γ-terpinene, linalool, p-cymene	Antibacterial agent, antioxidant	[19,23]
Tea tree	*Melaleuca alternifolia*	Terpenen-4-ol, γ-terpinene, 1,8-cineole, α-terpinene, cymene	Antibacterial agent	[19]
Citronella	*Cymbopogon nardus*	Citronellal, geraniol	Antibacterial agent, insecticidal agent	[15,19]
Cinnamon	*Cinnamomum zeilanicum*	Eugenol, cinnamaldehyde, linalool, geraniol	Antibacterial agent, antioxidant	[19,23]
Lavender	*Lavandula hybrida,* *Lavandula angustifolia*	Octyl acetate, linalool, camphor	Antibacterial agent, insecticidal agent	[15,19]

## 4. Application of Essential Oil Pickering Emulsions

As discussed earlier, essential oils show excellent properties, such as antioxidant, antimicrobial, and larvicidal activities, among others. To improve their effectiveness in delivering these beneficial properties when applied in various products, EOs are frequently present in the form of emulsions. In this context, the application of essential oil Pickering emulsions—ranging from drug carriers to active packaging—has been carried out in many studies, the results of which are tabulated in Table 2.

### 4.1. Essential Oil Pickering Emulsions as Drug Carriers

Application of essential oils in Pickering emulsion systems as drug carriers has been conducted using different essential oil sources, such as tea tree, lavender, anise, thyme, etc. Pickering emulsions composed of tea tree oils with chitosan nanoparticles as stabilizers show interesting results when applied to examine the wound-healing effects of their bioactive compounds. Pickering emulsions as carriers for the encapsulated tea tree oil result in a sustained-release effect and enhance the antibacterial effects against *Escherichia coli* and *Staphylococcus aureus*. When compared to classical emulsions (using Tween 80 as a surfactant), Pickering emulsions are more effective in delivering bioactivity as antibacterial carriers, which might be linked to the improved aqueous solubility of oil and the reduced degradation of the active ingredients [24]. In terms of stability, this Pickering emulsion system can stand for two months. Addition of curcumin into this Pickering emulsion leads to a better synergistic healing effect.

Another study on tea tree, thyme, and anise oil Pickering emulsions was conducted using silica nanoparticles as stabilizers. In terms of in vitro diffusion measured by estimating the cumulative amounts of essential oils penetrating the agar gel membrane, the Pickering emulsion displayed better results compared to conventional emulsions (using Tween 80 as a surfactant). This might be attributed to the smaller droplet size in Pickering emulsions [25]. Furthermore, the droplet size of the Pickering emulsion of tea tree oil was smaller than that of the anise and thyme oil Pickering emulsions. This shows that the type of oil determines the stability of the emulsion. Overall, Pickering emulsions display promise for applications as essential oil drug carriers, because the essential oils can be effectively transported to membrane complexes and are considered to be safer for the body than conventional emulsions that use surfactants as stabilizers.

### 4.2. Essential Oil Pickering Emulsions (EO-PEs) as Larvicidal Agent Carriers

A study on massoia and nutmeg essential oils showed that the components in the essential oils can function as larvicides against *Aedes albopictus* mosquitos, which play a role in transmitting the dengue, Chikungunya, and Zika viruses. The essential oils from massoia and nutmeg demonstrated high larvicidal activities—approximately 95% and 85% at 50 µg/mL, respectively. Amongst the compounds identified in massoia essential oils, benzyl salicylate, terpinolene, C12 massoia lactone, sabinene, benzyl benzoate, methyl eugenol, and C10 massoia lactone displayed the strongest larvicidal properties. When these essential oils are prepared in the form of Pickering emulsions using cellulose nanocrystals (CNCs) as stabilizers (with CNC concentrations of 270 mg/mL for massoia oil and 180 mg/mL for nutmeg oil), it was found that the larvicidal effects were stronger than that in the crude extract. This strong effect may be due to the improved aqueous solubility, longer shelf life, and more controlled release of the essential oils when prepared in the form of Pickering emulsions. These emulsions are also stable for at least 10 days with a droplet size of 3–15 µm [26].

Similar findings on the effectiveness of essential oils as larvicidal agents in Pickering emulsions compared with their pure forms are also presented in another study on white thyme essential oil [27]. The higher solubility and sustainability of essential oils in water may play a significant role in this effectiveness. Using CNCs as stabilizers, this Pickering emulsion was stable until 25 days with a droplet size of about 5 µm. In general, the size of the droplets decreases with the increase in the CNC concentration.

### 4.3. Essential Oil Pickering Emulsions as Antioxidant Carriers

Studies on the application of Pickering emulsions as antioxidant carriers have been conducted on cinnamon essential oil, pomegranate seed oil, and cedarwood essential oil. Application of Pickering emulsions of these essential oils can overcome their poor oxidative stability during storage.

Cinnamon-oil-based Pickering emulsions with breadfruit starch nanoparticles demonstrate good oxidative stability after 7 days of storage, with essential oil retention of approximately 79%. These Pickering emulsions possess initial droplet sizes ranging from 11 to 23 µm, increasing to 20–25 µm after 7 days of storage. Concentrations of 0.5–1% cinnamon oil show a higher loading efficiency than that at 0.1% [28].

Another study on the effects of Pickering emulsions on essential oils’ oxidative stability was performed using pomegranate seed oil (containing 16 fatty acids) and whey protein isolate (WPI) as a stabilizer. In this study, WPI was heated to create a microgel structure and then applied to stabilize the Pickering emulsion. This system was compared to an emulsion containing WPI in its natural form and to one containing commercial modified starch. Overall, pomegranate seed oil encapsulated in an emulsion system exhibited better stability against oxidation, with fatty acid retention ranging from 52 to 68% after 30 days of storage, depending on the fatty acid type. This system was also effective in protecting the oil against the formation of volatile compounds such as heptanal, (E,E)-2,4-heptadienal, (Z)-2-heptenal, octanal, pentanal, etc., compared to the free pomegranate oil. However when compared with the other emulsion types, the Pickering emulsion stabilized with WPI microgel displayed a higher droplet size (6.49–9.98 μm) compared to WPI (1.88–4.62 μm) and modified starch–WPI emulsions (1.68–5.62 μm) [29].

The effectiveness of Pickering emulsions in improving oils’ oxidative stability has also been compared to nanoemulsions. Cedarwood oil (CEO) was emulsified using Tween 80 and Span 80 as emulsifiers to form a nanoemulsions, while in the Pickering emulsion the oil was stabilized with OSA-modified starch. The results indicated that CEO in a nanoemulsion exhibits higher antioxidant activity than CEO in a Pickering emulsion [30], suggesting the effect of particle size on the antioxidant activity. However, compared to the pure CEO, both the nanoemulsion and the Pickering emulsion possesses higher free radical scavenging activity and iron-reducing power. This may be related to the poor solubility of pure CEO in water, preventing it from reacting rapidly and effectively.

### 4.4. Essential Oil Pickering Emulsions as Antimicrobial Agents

Essential oils (EOs) have excellent properties for food preservatives, such as antimicrobial activity, and are also listed as GRAS (generally recognized as safe) ingredients. Previous studies on the antibacterial and antifungal properties of cinnamon essential oils (in vitro and in vivo)—showing antifungal activity against *Rhizopus nigricans*, *Aspergillus flavus*, and *Penicillium expansum*, as well as antibacterial activity against *Paenibacillus larvae*—have been carried out [42,43]. These studies demonstrate that cinnamon oils display potent antimicrobial properties. However, decomposition and evaporation easily take place when exposing essential oils to light, oxygen, and heating in food processing [44], leading to limitations on their application in food preservation.

In order to improve the stability of these essential oils, Pickering emulsions can be used to protect them from environmental factors. Pickering emulsions can also prevent destabilization processes such as coalescence and Ostwald ripening better than traditional surfactants [45]. Several studies have reported that colloidal stable emulsions have higher resistance to the evaporation of volatile compounds than surfactant-stabilized emulsions, meaning that emulsions stabilized with solid particles (so-called Pickering emulsions) can maintain a sustained-release effect for the encapsulation of EOs [31].

#### 4.4.1. Emulsification of Cedarwood Essential Oil (CEO) by OSA-Modified Starch Pickering Emulsions

The poor water stability and strong volatility of cedarwood essential oil limit its applications in food, pharmaceutical, and cosmetic products. Emulsification of cedarwood essential oil by Pickering emulsions using OSA-modified starch can be used to solve the problem of solubility of the EO and is also necessary to maintain its bioactivities. Fifty-one components can be found in cedarwood essential oil, of which the most abundant are α-cedarene (32.72%), β-cedarene (12.26%), and thujopsene (24.03%). These compounds have bactericidal, insecticidal, and anti-inflammatory activities. At different ranges of solid particles used as stabilizers, 1% OSA-modified starch shows the formation of high-quality emulsions due to its small particle size and unimodal distribution.

Cedarwood essential oil in Pickering emulsion systems results in stronger antibacterial activity compared to that in pure form without emulsification, and even compared to that in nanoemulsion systems. This may be due to the fact that pure CEO has low water solubility, which hampers its interaction with the cell membrane. However, in emulsion systems this interaction can take place. Furthermore, the antimicrobial effectiveness of CEO-PE was found to be better than that of CEO-NE. This may be due to the negative charge of the surface of microorganisms and that of the emulsion. The greater electronegativity of CEO-NE droplets compared to CEO-PE droplets repels the microorganisms more strongly as they approach, leading to a weaker antibacterial effect [30].

#### 4.4.2. Emulsification of Cinnamon Essential Oil (CEO) by Zein–Pectin (ZP) Composite Nanoparticles

Zein–pectin-based nanoparticles are considered to be edible natural materials that are environmentally sustainable, chemically stable, and low-cost [31]. Research conducted by Zhou et al. [46] reported that solid zein–pectin particles can stabilize corn oil with a high internal emulsion.

A study on the emulsification of cinnamon essential oil with zein–citrus pectin as a stabilizer was conducted by Jiang et al. [31]. The results showed that the zeta potential of the cinnamon essential oil emulsion was 31.23 mV, suggesting a good stability of the emulsion. Meanwhile, the zeta potential of zein-alone nanoparticles was positively charged. The wettability value of the tablet that was immersed in CEO was 89.2°, indicating medium wettability. Several studies have reported that when the value of the three-phase contact angle is close to 90°, it is beneficial to the stabilization of the emulsion [47]. Zein-alone nanoparticles can stabilize a variety of oils but cannot stabilize cinnamon essential oil.

TEM analysis reveals that zein coats the citrus pectin in the shape of egg white and then forms a core–shell structure, leading to better stabilization. Generally, citrus pectin prevents direct contact between zein and the essential oil, limiting the zein’s flocculation.

Fumigation experiments were conducted to observe the inhibitory effects of zein–citrus cinnamon Pickering emulsions against *Alternaria alternata* and *Botrytis cinerea*. The emulsion gradient used in this study ranged between 0.04 and 0.16 µL/mL CEO. The growth of *A. Alternate* decreased with the increase in the essential oil’s concentration from 0.04 to 0.12 µL/mL. Higher concentrations of the EO did not significantly inhibit the growth.

Study of zein–citrus composite Pickering emulsions showed that the growth inhibition against *B. cinerea* was not significant in the first 48 h, except at the concentration of 0.04 µL/mL. SEM revealed that zein–citrus composite Pickering emulsions can destroy the integrity of cell membranes, which may lead to the death of the microorganism.

#### 4.4.3. Emulsification of Thymol with Zein/Gum Nanoparticle (ZGP)-Stabilized Pickering Emulsions

Thymol is an antimicrobial compound that has been proven to inhibit the growth of both Gram-positive and Gram-negative bacteria, such as *Escherichia coli*. The phenolic hydroxyl group of thymol can penetrate the lipid layer of microorganisms and act as a bactericide, which is beneficial in food and medicinal applications [48,49]. Thymol is easily degraded, has low water solubility, and has short-term bioactive availability; therefore the application of the product is limited. These poor properties can be improved by designing good delivery systems and controlling release using a particle-stabilized emulsion—the so-called Pickering emulsion.

Zein is a corn prolamine that has emulsifying ability due to 50% residues of hydrophobic surface amino acids. Zein-stabilized Pickering emulsions form droplet aggregates in emulsions; however, combining zein and other prolamine-containing amphiphilic groups can increase the stabilization. Gum Arabic (GA) is an amphiphilic polysaccharide. Therefore, combining zein and GA as nanoparticles to stabilize the emulsion can help to improve the emulsion stability. Research by Chen and Zhong [50] reported that zein can adsorb GA in a wide pH range via electrostatic and hydrophobic interactions; thus, zein–GA Pickering emulsions show better stability.

Research conducted by Li et al. on the use of zein–GA nanoparticles to stabilize thymol [34] showed that the creaming index significantly increased in the first 2 h of observation. The creaming index tended to stabilize after monitoring for 4 h. Increasing the concentration of ZGPs can significantly decrease the creaming index value (*p* > 0.05) and the viscosity. Therefore, the addition of ZGPs effectively inhibits the aggregation of the oil granules and results in stronger stability of the oil–water interface through spatial hindrance [51]. The droplet size of the emulsion is influenced by the different concentrations of ZGPs. The emulsion exhibited flocculation and agglomeration with the use of 1.25% ZGPs. Higher concentrations of ZGPs led to smaller particle sizes and gradual distribution. Emulsification of thymol with ZGPs can inhibit the growth of *E. coli*, and exhibits a controlled-release effect on thymol, and maintains antibacterial activity due to the stable interfacial layer protecting the thymol.

#### 4.4.4. Emulsification of Cinnamon Essential Oil with Nanocellulose Pickering Emulsions

Nanocellulose is a sustainable nanomaterial due to its abundant availability, biocompatibility, and eco-friendliness, as well as its surface chemistry and morphology. Different sizes and morphologies of nanoparticles can change the aspect ratio, rheology, capillary forces, and interface configuration of the emulsion [52]. Cellulose nanocrystals (CNCs) and nanofibers (CNFs) are good nanoparticles for stabilizing interfaces with different polarity due to their amphiphilic and anisotropic properties. Only low concentrations of CNCs and CNFs are required to form stable emulsions with high viscosity [53].

Emulsions’ stability is affected not only by the solid particles’ properties but also by the essential oil and the preparation method. A study investigating cellulose microfibers (CMFs) as solid particles for stabilizing emulsions with different *o/w* ratios found that a small *o/w* ratio formed a separate oil phase on the top of the emulsion, leading to an unstable emulsion [54]. Furthermore, Yu et al. [55] reported that application of CNCs for stabilizing clove oil emulsions produced high-stability emulsions until coalescence; nevertheless, the emulsions underwent gravity destabilization, i.e., flocculation and creaming, indicating the poor surface coverage at the oil–water interface.

A study by Alana Gabrieli de Souza et al. [33] on emulsions of cinnamon essential oil stabilized by CNC and CNF Pickering emulsions found particle sizes ranging from 25 to 50 µm and from 30 to 100 µm, respectively. Both CNC and CNF emulsions were stable after 30 days of storage, after which the CNC emulsion formed a cream layer at the bottom of the emulsion, with the most stable emulsions showing a 34.3% and 42.8% creaming index. The CNF emulsion showed no separation after 30 days of storage, due to its gel-like features and the CNFs self-assembling characteristics.

Both CNC- and CNF–based emulsions showed inhibitory activity against Gram-negative and Gram-positive bacteria, indicating the antimicrobial properties of the emulsion systems. The inhibitory value of the CNC emulsion (30.1) against *Bacillus subtilis* was lower than that of the CNF emulsion (65).

#### 4.4.5. Emulsification of *Rosmarinus officinalis* Essential Oils with Chitosan–Benzoic Acid Nanogels

Rosemary—or *Rosmarinus officinalis*—is an aromatic herb that has antimicrobial activity against *Salmonella* sp. Its encapsulated essential oil has proven to possess high bioactivity through activation of the passive cell adsorption mechanism [56]. EOs’ properties can be maintained and even improved using nanogels, which have been demonstrated to have high loading capacity, controlled-release properties, and high stability [57]. A commonly used food matrix in nanogels’ preparation is chitosan. This natural polysaccharide can be modified through the modification of hydrophobic moieties for nanogel applications [57]. Several previous studies have reported that chitosan is effective as a food matrix to suppress microorganisms’ growth and maintain food quality [57,58].

A recent study produced nanogels with the formation of amide linkages between chitosan amino acid and benzoic acid carboxyl groups to improve the antimicrobial activity and maintain the stability of the *Rosmarinus officinalis* essential oil (REOs) [36]. The formed gel nanoparticles exhibited a spherical shape, uniform size distribution, and mean diameter of 100 nm. Testing the antibacterial activity of REO incorporated in chitosan–benzoic acid nanogels showed that REO in emulsion systems is more effective in inhibiting the growth of *Salmonella tryphimurium* on beef cullet compared with non-emulsified REO. No significant change in color during storage was observed. Therefore, encapsulation of *Rosmarinus officinalis* can suppress the evaporation and instability of essential oils.

#### 4.4.6. Emulsification of Citronella Oil with Composite Microcapsules

Citronella oil (CTO) is obtained from the extraction of *Cymbopogon nardus*, which has been used in traditional Chinese medicine. This compound exhibits herbicidal, insect-repellent, and antimicrobial properties. The delivery of active ingredients in microcapsules has been studied to protect the bioactivity of citronella oil from environmental factors and control the release of its encapsulated bioactive materials [35]. The composite microcapsules use HAP or hydroxyapatite/quaternary ammonium salt of chitosan (HACC)/sodium alginate (SA) as a shell. Preparation of the microcapsule composites entails electrostatic adsorption of HACC and SA, followed by the chelation of alginate and Ca^2+^.

SEM analysis showed that the microcapsules’ shape was spherical. The release activity of the microcapsules was sustained throughout the observation. The system of CTO-loaded microcapsules exhibited antimicrobial activity against *Staphylococcus aureus* and *Escherichia coli* [35].

#### 4.4.7. Emulsification of Peppermint Oil with Composite Microcapsules

Peppermint oil is widely used in traditional Chinese medicine due to its unique functional properties, such as antibacterial, antifungal, antiviral, and anti-inflammatory activity. However, peppermint oil is a volatile compound that becomes chemically unstable in the presence of oxygen, light, and heat [59]. Microcapsules can protect oil molecules from contact with the external environment [60].

Peppermint oil encapsulated in a Pickering emulsion using chitosan-decorated silica nanoparticles (CSNs) has been studied previously [37]. CSNs can improve the hydrophobicity, leading to easy adsorption at the oil-in-water interface of Pickering emulsions. Moreover, the Pickering-emulsion-based composite microcapsules can prolong the stability and antibacterial activity of peppermint oil.

A Pickering emulsion of peppermint oil (PE-PO) stabilized with CSNs was prepared using the homogenization method. In this method, the ratio of oil to water was 1:9. Then, peppermint-oil-loaded composite microcapsules were prepared using a mini spray-dryer. Hydroxypropyl methylcellulose (HPMC) was used as the wall material. It was found that particle size decreased with the increase in the CSN concentration in the PE-PO. After 24 h, the PE-PO/0.5%CSN and PE-PO/1%CNS were prone to creaming. This means that 0.5% and 1% CSN could not stabilize the Pickering emulsions. PE-PO/2% did not exhibit creaming within 24 h. This could be attributed to the 2% CSN particles efficiently adsorbing onto the oil–water interface of the Pickering emulsion droplets. Based on the prolonged release, acceptable entrapment efficiency (89.1%), and drug loading (25.5%), the peppermint-oil-loaded composite microcapsules with 100% HPMC as the wall material (POCM-100%HPMC) appeared to be the optimal formulation. POCM-100%HPMC could significantly increase the PO’s stability. Furthermore, even after 60 days of storage, the POCM-100%HPMC had long-term antimicrobial activity (85.4%) against *S. aureus* and *E. coli* [37].

#### 4.4.8. Emulsification of Cinnamon Oil with Composite Microcapsules

Cinnamon oil (CMO), derived from cinnamon bark, leaves, and branches, has long been recognized as a natural antibacterial agent with broad-spectrum antimicrobial activity against the growth of bacteria, molds, and yeasts. CMO has some undesirable properties, such as easy volatility and a high vulnerability to oxygen, light, and heat, which significantly reduce its shelf-life and stability and shorten its functional activity efficiency, resulting in clear limitations on its practical application. Therefore, coating CMO in microcapsules is a promising strategy for addressing CMO’s easy volatility and high vulnerability [61,62].

Based on the findings of [32], the fabrication of CMO-loaded microcapsules could be achieved using the Pickering emulsion template. Because of its simplicity and versatility, the Pickering emulsion template approach has received a lot of attention for microcapsule fabrication. CMO-loaded antibacterial composite microcapsules were prepared using controlled-release properties and silicon dioxide (SiO_2_)/poly (melamine formaldehyde) (PMF) hybrid shells based on templating oil-in-water (*o/w*) Pickering emulsions. The stabilizing compound of the Pickering emulsion was SiO_2_ nanoparticles. Specifically, SiO_2_ nanoparticle suspensions were used as the water phase, while CMO served as the oil phase. The oil: water phase ratio was 1:4. The CMO-loaded composite microcapsules were prepared using in situ polymerization of CMO Pickering emulsion droplets. As a result, the diameter of the SiO_2_ nanoparticles was about 22 nm. The zeta potential of the SiO_2_ nanoparticles was −24.1 mV. Because the SiO_2_ nanoparticles were negatively charged, they could form electrostatic repulsion between nanoparticles to some degree, allowing for the stable dispersion of the SiO_2_ nanoparticles in water. The antibacterial properties of the CMO-loaded composite microcapsules were investigated via the inhibition zone method using *E. coli* and *S. aureus* as model bacteria.

The CMO-loaded composite microcapsule samples produced visible inhibition zones and, thus, demonstrated excellent antibacterial activity after 14 days. These results indicate that the CMO-loaded composite microcapsule samples have long-term antimicrobial activity.

### 4.5. Essential Oil Pickering Emulsions as Active Packaging

Today, as consumers attach more importance to replacing petroleum-based plastics, increasing concerns about environmental issues of plastic packaging waste, and food safety to protect humans from foodborne diseases, research on innovative bio-based active packaging materials is receiving significant attention. Biopolymers used in the production of biodegradable packaging materials include proteins, polysaccharides, and their combinations [33]. There are two types of active packaging methods in general: the first is the chemically active (chemical) method, and the second is the biologically active (biological compound) method. The latter is preferable because it is non-toxic, biodegradable, biocompatible, and long-lasting [63]. Concerns about the safety of commonly used chemically active agents also drive the demand for safer bioactive agents. Plant essential oils are generally considered to be safe, and they have powerful antioxidant and antibacterial properties, making them suitable for use in active packaging [64,65]. Plant essential oils, on the other hand, have limitations due to their high volatility, oxidation sensitivity, and the low water solubility of their active ingredients [66]. To address this issue, the Pickering emulsion method is thought to be a viable solution that can prevent essential oils’ evaporation and oxidation [67].4.5.1. Clove Essential Oil Pickering Emulsions and Gelatin/Agar Bio-based Films as Active Packaging

#### 4.5.1. Clove Essential Oil Pickering Emulsions and Gelatin/Agar Bio-Based Films as Active Packaging

In research by Roy and Rhim [38], gelatin was used as a bio-based packaging material. However, gelatin has some undesirable characteristics, including high hydrophilicity, low vapor-barrier properties, and lack of functionality. The best approach to solve the limitations of gelatin is to mix it with other polymers or to use a functional reinforcing filler. The combination of gelatin and agar is expected to result in a film with improved physical properties. Previously, in research by Mohajer et al. [68], these two polymers were used to create gelatin/agar-based binary composite films, and the results demonstrated that these two biopolymers have excellent compatibility with one another. Clove essential oil was used as a bioactive compound in active packaging due to its antimicrobial, antioxidant, and non-toxic properties. To overcome the limitations of clove essential oils, the Pickering emulsion method was used to prevent the essential oils’ evaporation and oxidation. Nanocellulose was used as a particle to stabilize the clove essential oil Pickering emulsions, due to its high physical stability, low cost, and eco-friendliness [69]. As a result, the particle size and zeta potential value of the clove essential oil Pickering emulsions was 60–120 nm, while the zeta potential was −51.8 mV. The strength of the film was affected significantly by the addition of clove essential oil Pickering emulsion (PEC). When a low concentration of PEC was added, the tensile strength increased significantly and then decreased linearly with the increase in the PEC concentration. The stiffness of the gelatin/agar-based film also decreased linearly with the increase in the PEC concentration. However, the flexibility of the film increased linearly with the increase in the PEC concentration. The addition of PEC significantly increased the antioxidant activity of the gelatin/agar-based film, with the degree of increase determined by the PEC concentration.

#### 4.5.2. Clove Essential Oil Pickering Emulsions and Chitosan Films as Active Packaging

In research by Xu et al. [39], chitosan was applied as an edible film because of its excellent film-forming performance and a certain degree of antibacterial characteristics. However, pure chitosan film has limitations due to poor water resistance. To improve the characteristics of edible chitosan films, chitosan was blended with clove essential oil (CLO) and encapsulated into oil-in-water emulsions. Zein colloid particles stabilize Pickering emulsions due to their high content of non-polar amino acids, endowing them with hydrophobicity. As a result, the particle size of Pickering emulsions with low concentrations of zein colloid particles was significantly larger than that of other emulsions. The zeta potential of all of the emulsions was above 40 mV. Typically, an absolute value of zeta potential greater than 30 mV is thought to be sufficient for emulsion systems to maintain their stability [70]. The thickness of the films was significantly increased by the addition of a CLO Pickering emulsion from 0% to 0.6%, going from 54.275 µm to 76.825 µm. The tensile strength of the films decreased with the increase in the clove essential oil, from 49.9 MPa to 30.4 MPa. The antimicrobial activity of CLO-Pickering-emulsion-containing edible films against *E. coli* and *S. aureus* was investigated. As expected, films incorporating a CLO Pickering emulsion had stronger antimicrobial activity than pure chitosan film, and the proportion of the CLO Pickering emulsion contributed significantly to the antimicrobial activity.

#### 4.5.3. Oregano Essential Oil Pickering Emulsions and Cellulose Nanofibrils as Active Packaging

In research by Wu et al. [40], cellulose nanofibrils (CNFs) were used as bio-based materials to prepare packaging films due to their mechanical properties and sustainability. CNFs were blended with oregano essential oil (OEO) Pickering emulsions using ZnO nanoparticles as stabilizers. Using ZnO nanoparticles to stabilize Pickering emulsions is a good way to encapsulate antimicrobial agents from essential oils and further enhance their antimicrobial activity in the developed films. As a result, the particle size of oregano essential oils was between 15 µm and 60 µm. The tensile strength of the films decreased with the addition of OEO Pickering emulsions. Despite the fact that the addition of Pickering emulsions had a significant impact on the tensile strength, the prepared film still had good mechanical properties. The antimicrobial activity of CNF films against *Listeria monocytogenes* was investigated. The CNF films were endowed with excellent antimicrobial and antioxidant activity after the addition of the OEO Pickering emulsions, as expected. The inhibition rate increased from 32.45% CNF film + 0.3 g of OEO Pickering emulsion to 89.61% CNFs film + 0.9 g of OEO Pickering emulsion, indicating that the functional ingredients in the Pickering emulsion were effective against *L. monocytogenes*.

#### 4.5.4. Oregano Essential Oil Pickering Emulsions and Soluble Soybean Polysaccharide as Active Packaging

In research by Liu et al. [41], soluble soybean polysaccharide (SPSS)-based films were fabricated that incorporated oregano essential oil (OEO) as an antimicrobial agent. OEO was encapsulated in a Pickering emulsion stabilized by complex coacervates of acid-soluble soy protein (ASSP). The advantages of SPSS include good emulsification capacity, excellent film-forming ability, and the provision of beneficial effects for health. As a result, the addition of OEO Pickering emulsions decreased the tensile strength from 15.73 mPa to 9.58 mPa. Because of the difficulty in lipophilic species forming a cohesive matrix, the presence of oil droplets decreased the tensile strength values of biopolymer-based films. Furthermore, films loaded with Pickering emulsions demonstrated a gradual decrease in their elongation-at-break values as the loading percentage increased. Because the loading of OEOs was obtained by embedding emulsion droplets into the continuous film matrix, the continuity and cohesiveness of the film were jeopardized; this phenomenon may be attributed to the reduced continuity of the films [71]. The films were tested for antimicrobial activity against three bacteria: *E. coli* O157:H7, *S. aureus*, and *Pseudomonas aeruginosa*. Except for the control, clear inhibition areas against the three microbial strains were observed after 2 weeks of storage for all Pickering-emulsion-loaded films.

## 5. Conclusions

Pickering emulsions have promising potential as vehicles for essential oils, due to their high stability, non-toxicity, and eco-friendliness. The Pickering emulsion system could be a solution for hydrophobic and easily damaged essential oils, making them easier to apply, providing a better effect on the resulting product, and preserving the benefits of their bioactive compounds. Pickering emulsions can be applied as vehicles for drug carriers, larvicidal agent carriers, antioxidant carriers, antimicrobial activity, and active packaging. Essential oil Pickering emulsions exhibit high emulsion stability and less sensitivity to evaporation and oxidation. They also demonstrate antimicrobial and antioxidant activity when used as microcapsules or active packaging. Overall, essential oils exert more beneficial activities when they are in the form of Pickering emulsions than in their pure form. This may be linked to the improved aqueous solubility, better oxidative stability, and controlled release of the essential oils.

## Figures and Tables

**Table 2:** Different applications of various EOs using different particle stabilizers in Pickering emulsion systems.

Application	Method					Results	Ref(s).
	Essential Oils	Particle Stabilizer	Particle Characteristics/Properties	Oil/Water Phase	Preparation	Pickering Emulsion Characteristics/Properties	
Drug carriers	Tea tree oil; Lavender oil; Clove oil; Olive oil; Corn oil	Chitosan nanoparticles (CS NPs)	Particle size: 300–600 nm; Zeta potential: +16 mV	Oil phase: tea tree oil with 100 microgram/mL curcumin; Water phase: 4 mg/mL CS NPs suspension; Oil/water ratio: 1/9	EO containing curcumin mixed with CS NPs suspension at concentrations of 2, 3, and 4 mg/mL; pH 7.4 with 4 mol/L added NaOH; Homogenization: ultrasonication	Zeta potential: +24 mV; Droplet size (at respective concentrations): 2.2, 1.9, and 1.7 µm; Viscosity (at respective concentrations): 1.18, 6.36, and 26.46 Pas; Rheology: shear thinning Stability: Good long-term stability (2 months)	[24]
Drug carriers	Anise oil; Tea tree oil; Thyme oil	Silica nanoparticle (native silica nanoparticle)/HS (hydrophilic silica), and modified silica (MET, ET, and Ph)) Tween 80	Particle size: 19.8–20.8 nm; Zeta potential: −116 to +83.1 mV	-	Homogenization: 13,500 rpm for 2 min (rotor–stator homogenizer)	The most stable emulsions were stabilized with particles modified by the ethyl group. Tea tree EO droplet size: 0.292–6.247 µm Thyme EO droplet size: 0.613–5.435 µm Anise EO droplet size: 1.393–5.362 µm	[25]
Encapsulated larvicides	Massoia and nutmeg essential oils	Cellulose nanocrystals (CNCs)	-	Aqueous phase: CNC solution (270 mg/mL CNC for massoia oil, 180 mg/mL CNC for nutmeg oil); Oil phase: massoia and nutmeg essential oils	Homogenization: ultrasonication at a 60% amplitude for 1 min using an ultrasonic processor	Droplet size: 3 to 15 μm	[26]
Encapsulated larvicides	White thyme essential oil	Cellulose nanocrystals (CNCs)	-	Aqueous phase: CNC solution (45, 90, 135, and 180 mg/mL EO); Oil phase: thymol white oil	Homogenization: ultrasonication at a 50% amplitude for 30 s	TSI values after 24 h for CNCs at 135 and 180 mg/mL: <12; TSI values for CNCs at 45 and 90 mg/mL: >50; Average shell size: 45 μm (45 mg/mL CNCs), 5 μm (135 and 180 mg/mL CNCs)	[27]
Antioxidant encapsulation	Cinnamon essential oil	Breadfruit starch nanoparticles	-	Oil phase: cinnamon oil (0.05, 0.1, 0.5, and 1% (*w/w*)) and dissolved in MCT oil; Aqueous phase: distilled water; Oil/water ratio: 40%:60%	Dispersed breadfruit starch (3 g) in distilled water with added oil phase; Homogenization: 10,000 rpm for 5 min using a rotor–stator homogenizer (T50 basic, IKA)	Droplet size: 11.66–23.62 µm (before storage) and 20.7–25.64 µm (after 7 days of storage); Viscosity: 629–813 cP (before storage) and 3209–5849 cP (after 7 days of storage); EI: 1 (before and after 7 days of storage)	[28]
Antioxidant encapsulation	Pomegranate seed oil (PSO)	Whey protein isolate (WPI) microgel; Modified starch (Capsul^®^) combined with WPI	-	Oil phase: pomegranate seed oil (PSO); Continuous phase: combination of WPI (8% *w/w*) and modified starch or WPI microgels (heated WPI); Oil/water ratio: 1:4	Homogenization: 18,000 rpm/10 min using a rotor–stator homogenizer (Ultra-Turrax IKA T18) Stability test: pH (2.0, 4.0, 6.0, 8.0), NaCl solutions (0.5, 1.0 and 2.0% *w/v*); Spray-drying stability test: 0, 7, 15, 21, 30 days at 40 °C	Zeta potential (under stress conditions/different pH values and NaCl concentrations): −63 to −37 mV; Zeta potential of reconstituted emulsion over time: −39 to −33 (WPI microgel), −55 to −45 (WPI), −41 to −33 mV (WPI: Capsul); Droplet size: 6.49–9.98 µm (WPI microgel), 1.88–4.62 µm (WPI), 1.68–5.62 µm (WPI Capsul). The average droplet size increased over time	[29]
Antibacterial microcapsules	Cedarwood essential oil (CEO)	OSA-modified starch	-	Particle concentration: 0.1, 0.5, 1, 2, and 3%; Oil phase: CEO 5% Aqueous phase: deionized water	Homogenization: 15,000 rpm for 20 min	Best formulation for CEO-PE with OSA-modified starch: 1%; Droplet size: 0.626 µm; Zeta potential: +27.58 mV; Viscosity: 1.0–1.3 mPas	[30]
Antibacterial microcapsules	Cinnamon essential oil	Zein–pectin composite nanoparticles	ZCP properties; Particle size: 660.8 nm; Zeta potential: +31.23 mV; Contact angle: 89.2°	Particle concentration: 2, 1.75, 1.5, 1.25, 1, 0.75, 0.5, 0.25, 0.125%; Oil phase: CEO; Aqueous phase: deionized water; Oil/water ratio: 1:1	Homogenization: 14,000 rpm for 4 min using a rotor–stator homogenizer	1% ZCPs while maintaining good physical stability. Above concentrations of 0.125%, emulsions stabilized by ZCPs were more stable than CP alone or zein alone	[31]
Antibacterial microcapsules	Cinnamon essential oil	Silicon dioxide nanoparticles	Average particle size: 22 nm (diluted particles 148.7 nm); Zeta potential: −24.1 mV	Particle concentration: 1, 2, 3, or 4%; Oil phase: cinnamon oil; Water phase: deionized water; Oil/water ratio: 1:4	Homogenization: 5000 rpm for 2 min using an FJ200S homogenizer	SiO_2_ nanoparticle concentration was 4 wt%; superfluous SiO_2_ nanoparticles were sedimented at the bottom of the CMO Pickering emulsion after standing for 2 days	[32]
Antibacterial microcapsules	Cinnamon essential oil	Cellulose nanocrystals (CNCs); Cellulose nanofibers (CNFs)	CNCs: Zeta potential: −33 mV; Length: 2 μm; Diameter 131 nm; CNFs: Zeta potential: −33.2 mV; Length: 4 μm; Diameter 66.3 nm	Aqueous phase: CNC or CNF solution; Oil phase: cinnamon oil; Particle concentration: 0.5 or 1%; EO concentration: 20 or 30%	Homogenization: 10,000 rpm or 12,000 rpm for 3 or 7 min	Zeta potential of CNCs: −29.3 to −28.3 mV; Zeta potential of CNFs: −18.3 to −11.1 mV; Droplet size of CNCs: 25 and 50 μm (without phase separation after 30 days); Droplet size of CNFs: 30 and 100 μm (with phase separation after 30 days); Best preparation: cellulose nanofibers, homogenization speed of 12,000 rpm, and oil concentration of 20%	[33]
Antibacterial microcapsules	Thymol	Zein–gum Arabic nanoparticles (ZGP)	-	Aqueous phase: Zein/GA–thymol nanoparticle dispersion (6.25% *w/v*); Oil phase: soybean oil; Oil fraction: 0.3	Homogenization: 10,000 rpm for 3 min using a high-speed homogenizer	Droplet size: 27.95–69.98 µm; Most stable emulsion: ZGP at a concentration of 2.5% and oil fraction of 0.1. Stable oil–water interfacial layer was destroyed by a higher concentration of NaCl (>150 mmol/L)	[34]
Antibacterial microcapsules	Citronella oil (CTO)	HAP (Hydroxyapatite) nanoparticles; Quaternary ammonium salt of chitosan (HACC); Sodium alginate (SA)	HAP Particle size: 397.4 nm; HAP zeta potential: −12.8 mV	Aqueous phase: HAP (hydroxyapatite) nanoparticles dispersion (1.0%) and sodium alginate (SA); Oil phase: citronella oil; Oil/water ratio: 1:1, 1:2, 1:3, 1:4	Homogenization: at 10,000 rpm for 2 min	CTO-loaded microcapsules possess high thermal stability; the in vitro release study of CTO shows that the microcapsules have sustained release activity	[35]
Antibacterial microcapsules	*Rosmarinus officinalis* essential oils	Chitosan–benzoic acid	-	Oil phase: REOs; Aqueous phase: chitosan–benzoic acid nanogels	Homogenization: sonication at 70 kHz for 5 min	-	[36]
Antibacterial microcapsules	Peppermint oil (PO)	Chitosan-decorated Silica nanoparticles	CSNs; Particle size: 118.12–152.5 nm; Zeta potential (from chitosan concentration 0–5%): −41.8 to +42.5 mV; Contact angle (from chitosan concentration 0–5%): 39.4°–67.4°	Oil phase: peppermint oil; Water phase: CSN suspensions (containing water); Oil/water ratio: 1:9	Homogenization: 22,000 rpm for 1 min using a high-shear homogenizer and high-pressure homogenizer (30 cycles at 800 bar)	Particle size: PO-PE 0.5%: 6.61 µm; PO-PE 2%: 3.73 µm; PO-PE 2% did not cream during storage	[37]
Active Packaging	Clove essential oil	Cellulose nanofiber (CNF)	-	Particle concentration: 0.75%; Oil phase: clove oil; Water phase: CNF aqueous solution	Homogenization: 5000 rpm for 1 h using an Ultra-Turrax blender	Particle size: 0.06–0.12 µm; Zeta potential: −51.8 mV	[38]
Active Packaging	Clove essential oil	Zein	-	Aqueous phase: zein suspension; Oil phase: clove oil; Zein concentration: 2%, 3%	Homogenization: 12,000 rpm for 10 min	The particle size of the emulsion decreased from 1.73 μm to 1.40 μm when the concentration of zein increased from 2% to 3%, indicating that higher concentrations of zein were required to reduce the size of the oil droplets and stabilize the emulsion	[39]
Active Packaging	Oregano essential oil	ZnO nanoparticles	-	Aqueous phase: ZnO nanoparticle dispersion; Oil phase: Mixture of oregano essential oil and soybean oil (1:1 ratio)	Homogenization: high-shear homogenizer at a speed of 10,000 rpm for 6 min	Droplet size: 15 μm to 60 μm. When the concentration of ZnO nanoparticles was 1.5 wt% and the mass fraction of the oil phase was 20%, the Pickering emulsion with a particle size of 26.85 μm exhibited strong standing stability	[40]
Active Packaging	Oregano essential oil	Soluble soybean polysaccharide (SSPS); Acid-soluble soy protein (ASSP)	Particle size of ASSP/SSPS complexes: 162.1 nm	Oil phase: Oregano essential oil mixed with soybean oil; Aqueous phase: ASSP dispersion	Homogenization: Homogenizer at a speed of 6000 rpm for 1 min	Particle size: 0.811 to 1.896 µm; Zeta potential: −4.48 to −3.41 mV	[41]

## Data Availability

Not applicable.
